# Rotational atherectomy of calcified coronary lesions: current practice and insights from two randomized trials

**DOI:** 10.1007/s00392-022-02013-2

**Published:** 2022-04-28

**Authors:** Abdelhakim Allali, Mohamed Abdel-Wahab, Karim Elbasha, Nader Mankerious, Hussein Traboulsi, Adnan Kastrati, Mohamed El-Mawardy, Rayyan Hemetsberger, Dmitriy S. Sulimov, Franz-Josef Neumann, Ralph Toelg, Gert Richardt

**Affiliations:** 1 Cardiology Department, Heart Center Segeberger Kliniken, Bad Segeberg, Germany; 2 Cardiology Department, Heart Center Leipzig, University of Leipzig, Leipzig, Germany; 3 Cardiology Department, German Heart Center, Technical University of Munich, Munich, Germany; 4 Cardiology Department, Vivantes Wenckebach Hospital, Berlin, Germany; 5 Cardiology Department, Heart Center Freiburg-Bad Krozingen, Bad Krozingen, Germany

**Keywords:** Rotational atherectomy, Calcified coronary lesions, Complex percutaneous coronary intervention

## Abstract

With growing experience, technical improvements and use of newer generation drug-eluting stents (DES), recent data showed satisfactory acute and long-term results after rotational atherectomy (RA) in calcified coronary lesions. The randomized ROTAXUS and PREPARE–CALC trials compared RA to balloon-based strategies in two different time periods in the DES era. In this manuscript, we assessed the technical evolution in RA practice from a pooled analysis of the RA groups of both trials and established a link to further recent literature. Furthermore, we sought to summarize and analyze the available experience with RA in different patient and lesion subsets, and propose recommendations to improve RA practice. We also illustrated the combination of RA with other methods of lesion preparation. Finally, based on the available evidence, we propose a simple and practical approach to treat severely calcified lesions.

**Graphical abstract:**

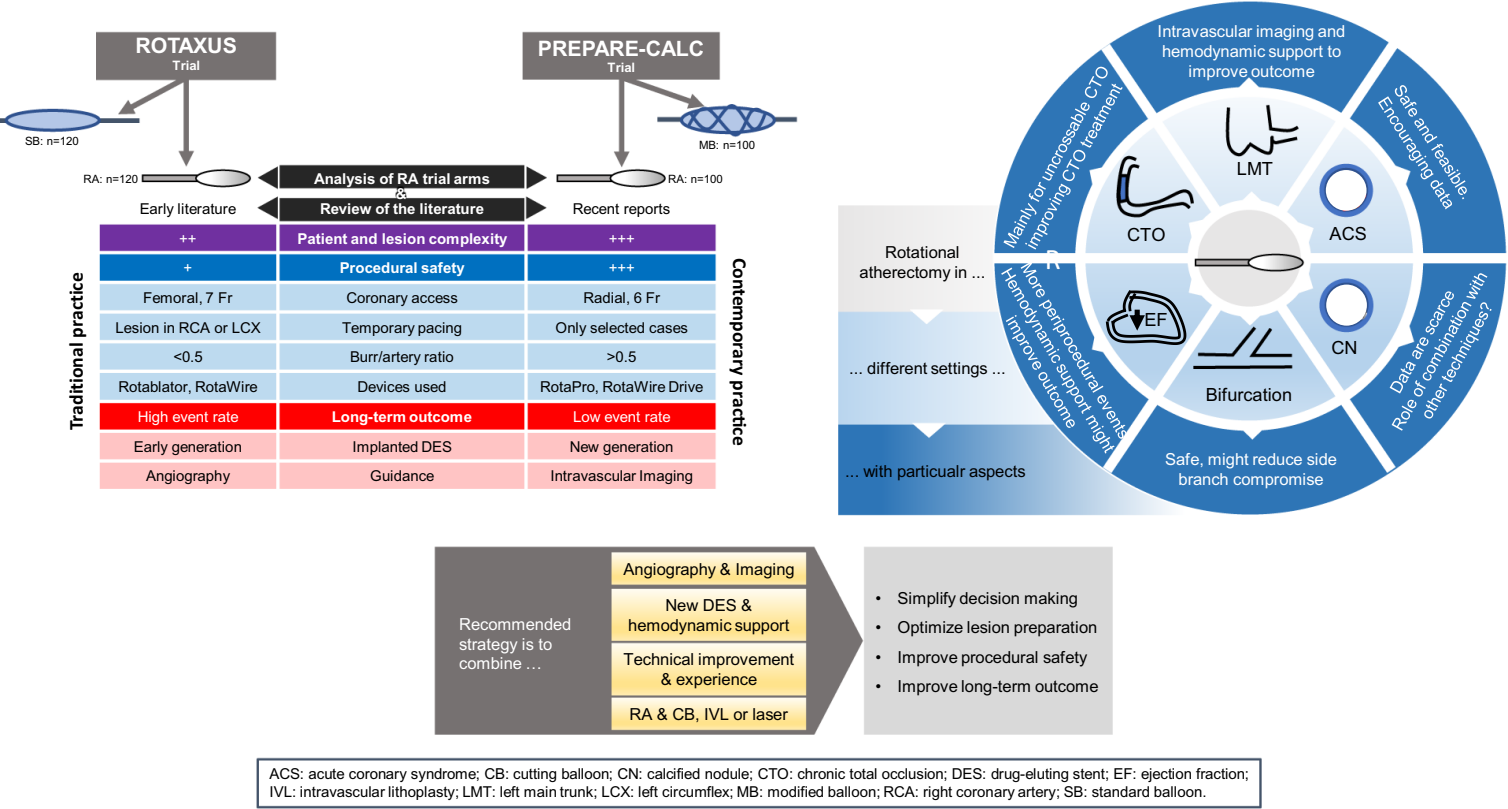

**Supplementary Information:**

The online version contains supplementary material available at 10.1007/s00392-022-02013-2.

## Introduction

In 1987, David Auth described**—**for the first time**—**rotational atherectomy (RA) as a new technique for endovascular treatment of atherosclerotic disease [1]. One year later, and after successful experience in animal models, Fourrier et al. reported the first human experience of percutaneous coronary RA in twelve patients [2]. Since then, the technique has been mainly preserved, and RA survived all developments of balloons and stents over the last four decades due to its unique properties in treatment of calcified lesions. Moreover, there are multiple reasons for a growing role of RA in the current era of coronary intervention.

First, the number of patients with severely calcified coronary lesions is rising due to an increasing prevalence of multiple risk factors, such as advanced age, diabetes mellitus and renal failure. Second, some technical refinements have improved the ease of use and the safety of RA. Third, there is consensus in most high-volume centers that certain complex calcified coronary anatomies cannot be properly treated without RA. Fourth, several observational studies and two randomized trials from the drug-eluting stent (DES) era, namely, the ROTAXUS (Rotational Atherectomy Prior to Taxus Stent Treatment for Complex Native Coronary Artery Disease) [3] and the PREPARE**–**CALC (Comparison of Strategies to Prepare Severely Calcified Coronary Lesions) [4] trials, have established more solid data on indications and outcomes of RA.

In this review, we summarize the current technique and indications of RA. This article also includes insights on the evolution of RA based on a pooled analysis of procedural and clinical data of the two available randomized studies in the DES era, which were conducted during an interval of one decade. Finally, we sought to provide a practical and systematic approach to treat calcified coronary lesions.

## Review methodology

Both ROTAXUS and PREPARE–CALC trials have been published in detail elsewhere [3, 4]. The ROTAXUS trial was an investigator-initiated, open-label, multicenter, randomized controlled trial that enrolled 240 patients with documented myocardial ischemia and moderate to severe calcified native coronary lesions undergoing percutaneous coronary intervention (PCI). Patients were enrolled in three high-volume interventional sites in Germany between August 2006 and March 2010 and randomized 1:1 to either RA or standard balloon (SB) dilatation followed by implantation of a polymer-based slow release paclitaxel-eluting stent (Taxus Liberté; Boston Scientific, Boston, Massachusetts). The primary endpoint of the trial was in-stent late lumen loss (LLL) at 9 months [3].

The PREPARE–CALC trial was performed between September 2014 and October 2017, and enrolled patients with documented myocardial ischemia and severely calcified native coronary lesions undergoing PCI. Two hundred patients with documented myocardial ischemia and severe calcification of the target native coronary lesion were enrolled in two high-volume centers in Germany, and were randomized 1:1 to an initial strategy of lesion preparation using RA vs. modified balloons (MB: scoring or cutting balloons) followed by implantation of a new-generation sirolimus-eluting stent with bioabsorbable polymer (Orsiro; Biotronik AG, Bülach, Switzerland). The primary endpoint of the trial was strategy success, defined as successful stent delivery and expansion with attainment of < 20% in-stent residual stenosis of the target lesion in the presence of TIMI (Thrombolysis in Myocardial Infarction) 3 flow without crossover or stent failure. The co-primary endpoint of the trial was in-stent LLL at 9 months [4].

In both studies, coronary angiography was digitally recorded and quantitatively assessed offline in an angiographic core laboratory (ISAResearch Center, Munich, Germany). Independent clinical event committees, blinded to treatment assignment, adjudicated all major adverse events in both trials. The studies were approved by the local ethics committees of all participating centers, and each patient provided written informed consent for inclusion in the respective analysis.

For the purpose of this review, data of patients randomized to RA (as per-protocol) in both ROTAXUS and PREPARE–CALC were pooled into a common database. We performed a comparison of clinical, angiographic and procedural characteristics of both RA arms. Results were displayed as number (percentage) for categorical data, and as mean ± standard deviation or median [interquartile range (IQR)] for continuous variables according to variable distribution. Student’s *t* test was used to compare normally distributed continuous variables and the Mann–Whitney *U* test was used for continuous non-normally distributed variables. The chi square and Fisher’s exact tests were used to compare categorical variables as appropriate. A two-sided *p*-value < 0.05 was considered significant. All statistical analyses were performed using STATA IC version 16.0 (StataCorp LCC, Texas, USA).

In addition, we performed a systematic review of the literature on RA by a PubMed search using following keywords: “Rotational atherectomy”, “Rotablation”, “Rotablator” and “RotaPro”. Observational studies, randomized trials and expert statements on RA were reviewed and considered when appropriate.

## Current technique of rotational atherectomy

### Patient characteristics and expertise of the operator

As shown in Tables 1 and S1, the RA-population in PREPARE-CALC was in average 4 years older than in ROTAXUS (74.3 ± 7.1 vs. 70.5 ± 8.2 years; *p* < 0.001) and patients were more likely to have chronic kidney disease (26% vs. 14%; *p* = 0.03). PREPARE–CALC RA-patients had more complex and extensive coronary artery disease. Lesion length was higher (29.81 ± 15.23 vs. 20.62; *p* < 0.01), and 10.6% of lesions were located in the left main trunk (LMT) vs. only 2.1% in ROTAXUS. These differences in clinical and angiographic characteristics can be explained by the difference in inclusion criteria of the studies. In the ROTAXUS trial, the main angiographic inclusion criterion was moderate to severe calcification (44.5% of severe calcification on core laboratory assessment) and LMT stenoses were excluded. PREPARE–CAC included only severely calcified coronary lesions (73% as adjudicated by the angiographic corelab) and LMT stenoses were not excluded, making the recent trial more reflective of the real word practice. Indeed, with the ageing of the population, the encouraging results of the contemporary DES trials and the progress in the available material and operator expertise, interventional cardiologists are more encouraged to treat the so called “Complex High-Risk Indicated Procedures/Patients: CHIP”. In this context, considering the excellent procedural and in-hospital outcome in PREPARE CALC, the study underscores the impact of the technical improvement and operator expertise on patient outcomes even in more complex lesion and patient subsets.

**Table 1:** Angiographic and procedural characteristics of both rotational atherectomy arms of PREPARE-CALC and ROTACXUS trials (*n* = 287 lesions)

	PREPARE-CALC (*n* = 141)	ROTAXUS (*n* = 146)	*p* value
Location			0.002
Left main	15 (10.6%)	3 (2.1%)	
Left anterior descending	78 (55.3%)	101 (69.2%)	
Left circumflex	16 (11.3%)	7 (4.8%)	
Right coronary artery	32 (22.7%)	35 (24.0%)	
Reference vessel diameter (mm)	3.25 ± 0.47	3.1 ± 0.4	0.001
Lesion length (mm)	29.8 ± 15.2	20.6 ± 9.3	< 0.001
Diameter stenosis (%)	83.0 ± 10.3	81.5 ± 10.2	0.198
Ostial location	40 (28%)	27 (18.5%)	0.05
Bifurcation	55 (39%)	72 (49.3%)	0.079
Moderate/severe tortuosity	49 (34.7%)	67 (45.9%)	0.054
Chronic total occlusion	4 (2.8%)	0 (0%)	0.057
B2/C lesion	137 (97.1%)	137 (93.8%)	0.175
7 Fr guiding catheter	130 (92.2%)	122 (83.6%)	0.02
Starting burr size (mm) Burr size (mm): 1.25 1.5 1.75 2.0	1.52 ± 0.17 24 78 32 4	1.51 ± 0.19 32 70 32 6	0.53
Max. burr size (mm)	1.53 ± 0.18	1.5 ± 0.2	0.77
Use of > 1 burr	7 (4.9%)	8 (5.5%)	0.74
Rotational speed (RPM)	164,224 ± 23,827	165,947 ± 8,919	0.89
Burr/artery ration	0.47 ± 0.07	0.50 ± 0.07	0.002
Balloon predilatation	119 (84.4.%)	130 (89.0%)	0.25
Max. predil. balloon diameter (mm)	2.97 ± 0.4	2.5 ± 0.3	< 0.001
Max. predil. balloon pressure (atm)	18.8 ± 3.7	13.6 ± 5.1	< 0.001
Predil. balloon/artery ratio	0.92 ± 0.10	0.8 ± 0.1	< 0.001
No. of stents/lesion	1.5 ± 0.6	1.3 ± 0.6	0.014
Total stent length/lesion (mm)	35.6 ± 15.7	27.7 ± 12.2	< 0.001
Min. stent diameter (mm)	3.1 ± 0.7	2.9 ± 0.4	0.0004
Max. stent diameter (mm)	3.3 ± 0.4	3.0 ± 0.4	< 0.001
Max. stent implantation pressure (atm)	16.5 ± 2.9	17.0 ± 2.9	0.108
Balloon postdilatation	111 (81.0%)	92 (63.0%)	0.003
Max. postdil. balloon diameter (mm)	3.7 ± 0.5	3.3 ± 0.5	< 0.001
Max. postdil. balloon pressure (atm)	20.9 ± 4.9	21.7 ± 5.8	0.28
Postdil. balloon/stent ratio	1.19 ± 0.15	1.13 ± 0.15	0.01
Pacing in RCA and LCX lesions (77 patients*)			
TP	18 (46%)	30 (79%)	0.003
No TP	21(54%)	8 (21%)	

Values are *n* (%) or mean ± SD

*RPM* rotations per minute; *TP* temporary pacing

*Patients with permanent pacemaker or implantable defibrillator were excluded

In an analysis of 30,268 CHIP–PCI procedures in England and Wales, Kinnaird et al. demonstrated that higher operator volumes were associated with a greater degree of patient comorbidity and increasing procedural complexity, but this was not translated into higher in-hospital and 12 months mortality [5].

### Vascular access and guiding selection

In general, treatment of complex calcified coronary lesions requires careful planning and anticipation of potential challenges. Several aspects need to be considered when guiding catheter and vascular access are selected. These include stability of the system during the procedure, sufficient internal diameter for the selected burr size, experience of the operator with radial and femoral access, and the risk of bleeding and vascular complications. Most procedures can be safely performed with a standard 6 Fr guiding catheter, which can accommodate burrs up to 1.5 mm. For a 1.75 mm burr size a 7 Fr guiding catheter is recommended, and for ≥ 2 mm burrs an 8 Fr guiding catheter should be considered. The inner diameter of guiding catheters may slightly vary among manufacturers, and RA with a 1.75 mm burr can be eventually performed through selected 6 Fr catheters. In the pooled data of the ROTAXUS and PREPARE–CALC trials, 70% of lesions have been treated using 1.25 and 1.5 mm burr sizes (Table 1). Notably, in the most contemporary trial, 7 Fr guiding catheters were used in 92% of lesions, probably due to the higher rate of left main lesions (10.6% vs. 2.1%; *p* < 0.002) and larger reference vessel diameter (3.25 ± 0.47 vs. 3.08 ± 0.40; *p* < 0.001), where operators anticipated an upgrade to larger burr sizes or a possible two-stent technique in case of bifurcations.

While procedures in both randomized trials were almost exclusively performed through femoral access, several recently published retrospective analyses reported the feasibility of RA through the radial access with similar procedural success and lower bleeding and vascular complications compared to femoral access [6–10]. With the availability of sheathless guiding catheters and slender sheaths with smaller outer diameters, RA even with large burr sizes can be safely performed via the radial artery.

Performing RA in a resistant lesion could usually solve back-up problems encountered during balloon and stent advancement. Nevertheless, in some cases of sub-optimal guiding support in very resistant lesions, back-up enhancement to perform RA becomes a necessity. To enhance guiding support during RA some simple tools such as the use of large guiding catheters, long sheaths and deep intubation could be used. Some more complex methods have been recently reported, and Mitomo et al. recently described the use of a buddy-wire during RA. After advancing the burr to the park position in the marginal branch, they advanced a supportive wire in the left anterior descending artery (LAD) through the side of the drive shaft sheath of the Rotablator™ to stabilize the guiding catheter during RA of the marginal branch [11]. RA through a guiding extension can also be performed to enhance guiding support particularly for the right coronary artery (RCA), or to treat distal lesions through recently implanted stents in the proximal part of the vessel.

### Temporary pacing

Due to the risk of distal embolization of calcific particles with microvascular ischemia and the resulting neuro-hormonal responses, the occurrence of transient heart block during RA was common, particularly as long as aggressive debulking was performed. As a result, temporary pacing was routinely recommended during RA of the RCA and dominant left circumflex (LCX) [12].

Comparing RA of the RCA and LCX in the two randomized trials, temporary pacing was less frequently performed in PREPARE–CALC compared to ROTAXUS (46% vs. 79%; *p* = 0.003) (Table 1). In a European expert consensus document on RA, routine use of transient pacing is no longer recommended. On one hand, there are concerns about prolonging the procedure and the risk of right ventricular perforation and tamponade associated with temporary pacing. On the other hand, heart block occurring during RA rapidly regresses or simply resolves with vagolytic maneuvers and can be prevented with atropine (0.5–1.0 mg IV) or aminophylline (250–300 mg over 10 min) [13, 14]. In contemporary practice and with modified techniques including less aggressive debulking, small burrs and avoidance of routine use of calcium antagonists, temporary pacing should be performed in few selected cases (e.g., severely reduced ejection fraction with last remaining RCA, or pre-existing high degree conduction abnormalities in an unstable patient).

### Burr selection and debulking technique

In both randomized trials, about half of the lesions were treated using a 1.5 mm burr, whereas 1.25 and 1.75 mm burrs were used in about 25% of cases, respectively (Table 1). The use of more than one burr was necessary in only 5% of cases. Of interest, a significantly lower burr-to-artery ratio was observed in PREPARE–CALC vs. ROTAXUS (0.47 ± 0.07 vs. 0.50 ± 0.07; *p* = 0.002), which documents the less aggressive ablation strategy that was adopted in contemporary practice.

**Fig. 1:**
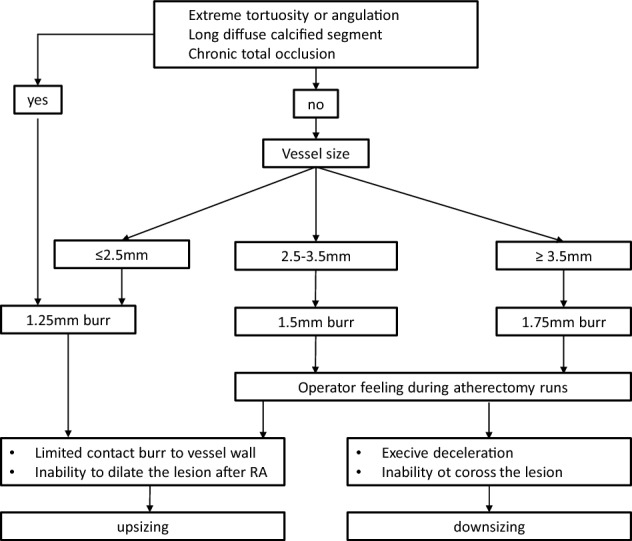
Burr selection for rotational atherectomy

In the much earlier CARAT and STRATAS trials, an aggressive debulking strategy (burr-to-artery ratio > 0.7) increased procedural complications without any advantage compared to a less aggressive lesion modification strategy [15, 16]. Burr selection depends on the vessel size and lesion morphology. For long very tight lesions, tortuous vessels and chronic total occlusions, a 1.25 mm burr is appropriate. In other cases, a burr-to-artery ratio of 0.5–0.6 should be considered. According to the operator assessment during burr runs, the burr size can be adapted. In case of high resistance with excessive decelerations during RA a downsizing is recommended. In contrast, if the lesion remains undilatable after RA or if the operator perceives no contact between the burr and the plaque an upsizing is preferable (Fig. 1). In all cases continuous rotation should be maintained during ablation runs in both advancement and pullback movements of the burr to avoid burr entrapment.

An important aspect of the debulking technique is the rotational speed [14]. A speed between 135,000 and 180,000 rpm is recommended by experts. Lower speed might cause burr lodging, and higher speed increases platelet activation and thrombotic complications [17, 18]. In our two randomized trials, the mean rotablation speed was 160,000 rpm. In clinical practice, operators may prefer increasing rotablation speed to cross resistant lesions. Recently, Yamamoto et al. evaluated low- following high-speed RA with high-speed RA in 30 patients using optical frequency domain imaging. Using the combination of low- and high-speed RA, larger minimal lumen diameters and a trend towards larger minimal lumen area were achieved compared to high-speed RA alone [19].

### Intravascular imaging and stent-optimization techniques

While debulking became less aggressive in the contemporary PREPARE–CALC trial, balloon predilation was performed with much more effort compared to the ROTAXUS trial. Significantly higher balloon pressures (18.8 ± 3.7 vs. 13.6 ± 5.1; *p* < 0.001) and balloon-to-artery ratio (0.92 ± 0.1 vs. 0.83 ± 0.1; *p* < 0.001) for balloon predilation were applied. Furthermore, to optimize stent results, postdilation was performed more frequently (81% vs. 63%; *p* = 0.003) and with higher balloon-to-stent ratios (1.19 ± 0.15 vs. 1.13 ± 0.15) in PREPARE–CALC (Table 1). This finding could be related to the use of optical coherence tomography (OCT) in the PREPARE–CALC trial in addition to the better understanding of the potential predictors of stent failure in contemporary practice.

Although the results of baseline OCT in PREPARE–CALC did not impact the choice of lesion preparation strategy, which was done according to randomization, OCT findings certainly helped the operators to select the burr and balloon sizes, and to achieve proper selection of stent length and diameter to avoid an incomplete lesion coverage, which is a predictor of stent thrombosis and restenosis. Following stent implantation, OCT can also detect correctable abnormalities, such as underexpansion, geographic plaque miss, strut malapposition and stent edge dissection. These abnormalities known to be associated with adverse PCI outcomes often lead to a reaction from the operator [20]. In a single center retrospective comparison of patients undergoing RA guided by OCT vs. IVUS, Kobayashi et al. found that OCT guidance resulted in more frequent burr upsizing, and larger final burr size and improved stent expansion [21]. Recently, Hemetsberger et al. reported the results of the OCT sub-study of the PREPARE–CALC trial [22]. Stent expansion was 74% and 73% (*p* = 0.85), and the cutoff of > 80%, as suggested by the current recommendations of intravascular imaging, was achieved in only 61% and 73% of cases in the MB and RA groups, respectively (*p* = 0.30) [20]. Of note, in 39% of patients, baseline OCT was not available. In the majority of these cases the OCT catheter could not cross the calcified lesion.

### RA in the era of new-generation drug-eluting stents

The advantages of DES over bare-metal stents following RA are well established [23]. Currently, after successful lesion preparation using RA, implantation of DES is considered the treatment of choice [24]. Although several trials reported encouraging long-term outcomes after RA [25–29], the rate of cardiovascular events and repeat revascularization remain relatively high in patients with calcified lesions, and the systematic use of RA did not improve long-term outcome as compared with a balloon-based strategy [30]. The major drawback of RA in previous trials was its association with an exaggerated neointimal response triggered by thermal injury [31, 32]. In the ROTAXUS trial, where an early generation DES (EG-DES) was implanted, LLL in the RA group was significantly higher compared with standard balloon predilatation [3].

**Fig. 2:**
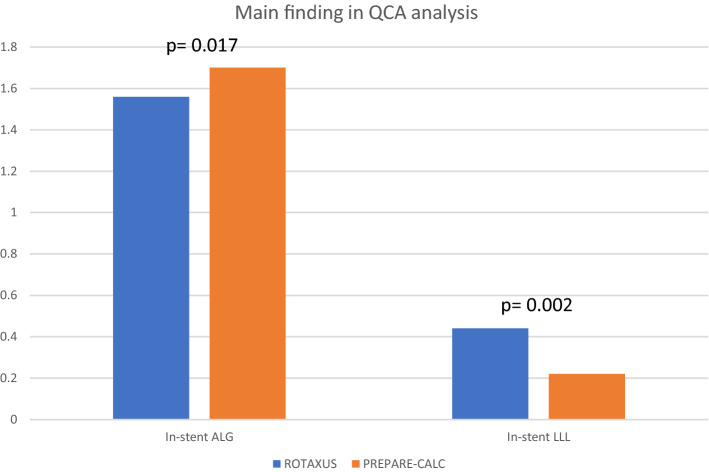
Main quantitative coronary angiography findings of rotational atherectomy arms form PREPARE-CALC and ROTAXUS trials. *ALG* acute lumen gain; *LLL* late lumen los; *QCA* quantitative coronary angiography

**Fig. 3:**
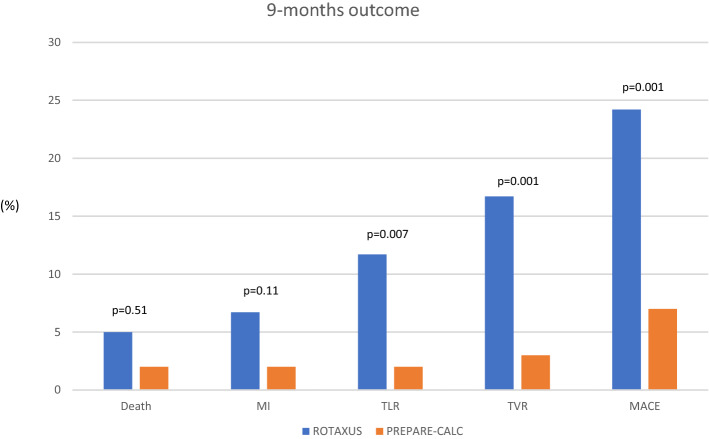
Nine months outcome of rotational atherectomy arms form PREPARE-CALC and ROTAXUS trials. *MACE* major adverse cardiovascular events; *MI* myocardial infarction; *PCI* percutaneous coronary intervention; *TLR* target lesion revascularization; *TVR* target vessel revascularization

New-generation DES (NG-DES) have more biocompatible anti-proliferative drugs and polymers supported by thinner struts, that led to better late clinical outcomes compared to EG-DES in a broad PCI population [33, 34]. Over the last few years, several case series have focused on long-term outcome after NG-DES with adjunctive RA (Table 2). Kawamoto et al. in their analysis from the multicenter ROTATE registry, found an advantage of NG-DES over EG-DES in terms of clinically driven MACE at 2 year follow-up [35]. From the same registry, another analysis demonstrated that the use of NG-DES seemed to be protective against MACE (HR 0.53, 0.31–0.88, *p* = 0.02). In another retrospective analysis of 481 RA patients, the comparison of EG- vs. NG-DES revealed lower mortality (13.5% vs. 8.2%, log-rank *p* = 0.13; adjusted HR after Cox regression analysis 0.49; 95% CI 0.26–0.92; *p* = 0.03) and MACE (31.1% vs. 21.1%, log-rank *p* = 0.04; adjusted HR 0.65; 95% CI 0.42–0.98; *p* = 0.04) at 2-year follow-up, despite more complex patients and lesions in the NG-DES group [36]. On the contrary, two other retrospective studies reported similar mid- and long-term outcomes between patients receiving NG- and EG-DES following RA [37, 38]. In PREPARE–CALC, RA followed by implantation of the NG Orsiro sirolimus-eluting stent (Biotronik) was not associated with excessive neointimal response, with no significant difference in LLL compared to predilatation with an MB [4]. A comparison of the two generations of DES following RA regarding LLL in the two randomized trials clearly reveals an advantage for NG-DES (0.22 ± 0.39 vs. 0.44 ± 0.58 mm; *p* = 0.006) (Fig. 2 and Table S2), which also translates into lower TLR (2% vs. 11.7%; *p* = 0.007) and MACE rates (8% vs. 24.2%; *p* = 0.001) at 9 months in the contemporary trial (Fig. 3). The role of ultra-thin stent platforms has been recently illustrated in a single-centre retrospective study by Mankerious et al. who compared the Orsiro biodegradable-polymer sirolimus-eluting stent vs. durable-polymer everolimus-eluting stent after RA. Although, clinical outcome was similar between the groups in the whole population, this study revealed significantly lower rate of target leison failure in a subgroup of patients treated with the ultra-thin Orsiro Stent (diameter ≤ 3 mm) [39].

**Table 2:** Published experience with RA and NG-DES

Author	Year	*N*	Follow-up (M)	MACE (%)	All cause death (%)	Cardiac death (%)	MI (%)	TVR (%)	TLR (%)	ST (%)
Allali et al. [36]	2018	NG-DES 213 EG-DES 268	24	21.1* 31.1	8.2 13.5	6.8 5.6	4.1 4.9	12.9 17.6	7.9 12.7	2.4 0.9
Hachinohe et al. [111]	2017	NG-DES 744	12	8.6	5.5	2.2	0.1	–	2.9	0.1**
Jinnouchi et al. [112]	2015	NG-DES 252	24	20.3	13.5	7	2.1	24.8	21.9	2.1
Tian et al. [38]	2015	NG-DES 59 EG-DES 40	12	11.9 12.8	8.5 10.3	8.5 2.5	0 0		3.6 2.7	0 0

*EG-DES* early generation drug-eluting stents; *MACE* major adverse cardiac events; *MI* myocardial infarction; *N* number of patients who received new generation DES; *NG-DES* new generation DES; *ST* stent thrombosis; *TLR* target lesion revascularization

*Statistically significant difference

**Definite ST

### Technical improvement

The new generation RotaPro system (Boston Scientific) has been designed to simplify the use of RA. The foot pedal has been replaced by three buttons for manual control on the advancer (high-speed, Dynaglide and to change between high-speed and Dynaglide). The console is digital with deceleration alerts and the system can be powered by either compressed air or nitrogen.

In 2021 Boston Scientific launched the new RotaWire Drive™ guidewire developed in collaboration with Asahi (Japan). The torque transmission and the kink resistance, which were the main limitations of the previous model, have been improved. The RotaWire™ Drive has an overall length of 330 cm and is available in two models: Floppy and Extra Support. The floppy wire with its longer flexible taper (13 cm) and shorter spring length (2.2 cm) reduces wire bias and is used in most cases. The extra-support wire has a shorter stiff taper (5 cm) with a longer spring (2.8 cm), causes more wire bias and is useful in distal lesions and aorto-ostial lesions.

## Complications of RA

Due to the growing experience with RA and by applying the correct technique, the rate of complications following RA became very low in recent publications. No/slow flow was reported in 2% in the PREPARE–CALC trial and 0% in ROTAXUS (Table S3). In the DES era, this complication occurs in 0–2.6% of cases [24, 40]. No/slow flow is the result of microvascular embolization of atherosclerotic debris together with platelet activation, thrombi and vasoactive reaction. Optimal antithrombotic therapy, flush cocktail and correct ablation technique are the key to avoid this complication. The key elements of optimal technique are the burr-to-artery ratio (0.4–0.6), the ablation speed (135,000–180,000 rpm), short ablation runs (< 30 s), gradual burr advancement with pecking motion and avoidance of deceleration of more than 5000 rpm [24]. In addition, correction of hemodynamic disturbance using fluids, vasopressors, pacing and if required hemodynamic support is very important particularly in patients with reduced ejection fraction.

The incidence of coronary dissection after RA varies from 1.7% to 5.9% in the DES era [40]. In PREPARE–CALC, large coronary dissections were numerically lower in the RA compared to the MB group (3% vs. 7%; *p* = 0.33)[4]. In a large registry, coronary dissections occurred more frequently when bailout RA was performed after an initial balloon strategy as compared with elective RA [41].

In our randomized trials, coronary perforation rate did not differ between RA and non-RA groups, and in the recent literature this complication occurs in 0–2% of cases [40]. Of note, in an analysis of 59 grade III coronary perforations from 24,465 PCI, the device causing perforation was a balloon in 50% of cases and RA in 3.6% [42].

Burr entrapment is a rare but serious complication, and its risk is higher in rotablation of freshly implanted underexpanded stents [43]. Retrograde ablation is not possible due to the absence of diamond chips on the rear of the burr, so carful, interventional maneuvers are needed to solve the problem (balloon dilatation proximal to the burr, deep guide intubation or guide-extension, dissection reentry with balloon dilatatation), but surgical intervention is required if conservative solutions fail [24].

## Patients and lesions treated with RA in the current practice

### Decision between elective or bailout RA (Fig. 4)

**Fig. 4:**
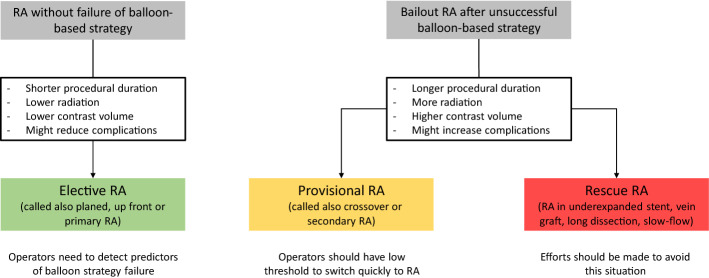
Elective and bailout rotational atherectomy. *RA* rotational atherectomy

In a randomized setting, up to 12.5% of patients from the SB group in the ROTAXUS trial and 16% from the modified balloon (MB) group in PREPARE–CALC crossed over to RA [3, 4]. In real live, experienced operators prefer performing elective RA in severely calcified and highly complex lesions. In the ROTATE registry, in nearly half of the cases RA was performed electively, and in the Bad Segeberg single-center registry, elective RA was performed in 60% of patients [41, 44]. Both analyses revealed that planned RA was associated with shortened procedural duration and reduction of peri-procedural events compared to bail-out RA. In fact, with operator experience and increasing confidence with RA technique, bail-out attitude tends to be replaced by a more elective indication to RA. In this context, the ideal scenario would be to define factors that predict failure or success of a balloon-based strategy. In the PREPARE–CALC trial, sub-group analysis suggests that RA would be more often required in males, in lesions located in the right coronary artery or left circumflex, severe (≥ 80%) stenosis and type C lesions [4, 45]. If in doubt, operators should have a low threshold for adopting an elective RA strategy or to switch quickly to provisional RA if an initiated balloon-based strategy is not successful. Rescue RA after long lesion manipulation with consequent long dissection and slow-flow, in a vein graft or in an underexpanded stent are situations with high risk of complications and efforts are needed to avoid these scenarios.

### Rotational atherectomy and left main disease

In patients with severe calcified left main trunk (LMT) lesions and prohibitive risk of bypass surgery, RA provides an effective alternative therapy, particularly in those patients with simple to moderately complex concomitant coronary artery disease. Furthermore, in patients with reduced ejection fraction or hemodynamic instability, RA offers a quick and effective solution to avoid multiple, prolonged, high-pressure balloon inflations to prepare resistant plaques located in a vessel with a large territory of perfused myocardium. In the contemporary PREPARE–CALC trial, RA was performed for left main disease in 10.6% of the treated lesions. Although LMT location was excluded in ROTAXUS, the rate of treated LMT was 2.1%, and in these cases the primary lesion was in the ostium of left anterior descending artery (LAD) or left circumflex (LCX) so that the LMT was involved during PCI.

The rate of LMT-RA to the total number of LMT PCIs and to the total number of RA varies among studies from 8.9% to 11.3% and from 6% to 9.3%, respectively [46–54]. The previously published experience with RA in LMT is summarized in Tables 3, 4. Overall, patients undergoing RA in LMT were older and had high prevalence of diabetes mellitus. Published cohorts reported unprotected LMT rotablation in 62% to 100% of cases, and in more than 65% the treated lesions involved the LMT bifurcation. Whereas LMT-RA resulted in satisfactory angiographic and in-hospital results, long-term outcome revealed relatively high incidence of cardiovascular events. Ielasi et al. reported in their analysis form the multicenter ROTATE registry a 12.9% mortality and a 14.7% TLR rate at 1-year follow-up, with significantly higher incidence as compared with non LMT RA [50]. Fuku et al. described in a recent analysis a TLR rate of 22% at 5 years [48], and in another cohort by Sulimov et al., a TLR rate of 17.3% was reported during a mean follow-up period of 2 years [49]. The incidence of stent thrombosis was 3.9% in the ROTATE LMT-RA, and Yabushita et al. reported a rate of 6.1% at 12 months follow-up [46]. It is possible that this is the result of the lack of intracoronary imaging guidance during PCI in the reported experience. It is worth mentioning here that a systematic intravascular ultrasound (IVUS) guidance in LMT PCI was found to be associated with a benefit in long-term outcome rather than an angiographic-only guided PCI [55]. Another important limitation is that the implementation of hemodynamic support did not exceed 20% in most reported series. Consequently, we can speculate that the high incidence of cardiovascular events comes from sub-optimal results that were accepted by the operator for the sake of terminating the procedure in a patient who may become unstable at a certain point of time. In summary, although RA seems to be feasible and safe in LMT lesions, intravascular imaging together with more frequent use of mechanical circulatory support may optimize stent results and possibly improve long-term outcome.

**Table 3:** Published experience with RA in LMT lesions (important patient and lesion characteristics)

Author	Year	*n* (%)	Age (years)	DM (%)	Previous CABG (%)	ULMT (%)	SYNTAX Score	Bifurcation (%)	Ostial (%)	Intravascular Imaging (%)	Circulatory support (%)
Dhillon et al. [47]	2019	55	72.9 ± 9.9	60	36.4	69	33.21 ± 8.9	76.4	na	IVUS 36	pLVAD 47.3 IABP 20 ECMO 3.6
Fuku et al. [48]	2019	108 (6%)*	75.7 ± 10.1	43	27	na	33.2 ± 10.3	69	na	IVUS 66 OCT 5	IABP 5
Ielasi et al., (ROTATE registry)[50]	2017	86 (8.9%)**	73.2 ± 7.5	50	5.8	100	30.1[21.7–36.6]	73.3	na	IVUS 55.3	IABP 10.5
Sulimov et al. [49]	2015	50 (11.3%)**	73 ± 8.1	42	50	62	28.6 ± 8.2	80	4	0	8%
Yabushita et al. [46]	2014	64 (9.3%)*	71.4 ± 8.7	54.7	Na	100	35.4	73.4	-	IVUS 73.4	IABP 12.5
Dahdouh et al. [51]	2013	13	83.1 ± 2.3	23	7.7	92.3	29.7 ± 7.5	na	23.1	0	0%
Chiang et al. [52]	2013	34	77.2 ± 10.2	52.9	29.4		50 ± 15	100	8.8	na	IABP 20
Garcia-Lara et al. [53]	2012	40	73 ± 8.3	55	Na	100	27.45 ± 9.9	67	52	IVUS 25	IABP 7.5
Schwartz et al. [54]	2011	31	75.5 ± 9.4	61	68	11	na	65	na	Not used	Not used

Data are presented in number or percentage, mean ± standard deviation or median [interquartile range]

*CABG* coronary artery bypass graft; *DM* diabetes mellitus; *IABP* intra-aortic balloon pump; *IVUS* intra-vascular ultrasound; *LMT* left main trunk; *OCT* optical coherence tomography; *pLVAD* percutaneous left ventricular assist device; *RA* rotational atherectomy; *ULMT* unprotected LMT

*% to the total number of LMT PCI

**%to the total number of RA

**Table 4:** Published experience with RA in LMT lesions (in-hospital and long-term outcome)

Author	Year	Patients	Angiographic success (%)	Peri-procedural MI (%)	In-hospital death (%)	Follow-up (months)	All cause death (%)	Cardiac death (%)	TLR (%)	ST (%)
Dhillon et al. [47]	2019	55	100	20 (Troponin > 3xbaseline)	3.6	12	na	na	10.9	na
Fuku et al. [48]	2019	108	na	6.4	2.8	60	34	14	22	1
Ielasi et al., (ROTATE registry) [50]	2017	86	na	5.8	1.2	12	12.9		14.7	3.9
Sulimov et al. [49]	2015	50	96	2	6	24	17.3	10.8	17.3	2
Yabushita et al. [46]	2014	64	95.3	7.8	0	12	9.4	6.3	18.8	6.1
Dahdouh et al. [51]	2013	13	92.3	16.7	7.7	25	8.3	8.3	25	
Chiang et al. [52]	2013	34	100	5.8	8.8	30.4	0	0	16.1	
Garcia-Lara et al. [53]	2012	40	na	na	7.5	24.7	32.5	29	19.3	na
Schwartz et al. [54]	2011	31	90	0	3	15.3	13		9	0

Data are presented in number or percentage, mean ± standard deviation or median [interquartile range]

*LMT* left main trunk; *MI* myocardial infraction; *ULR* upper limit range; *RA* rotational atherectomy; *ST* stent thrombosis; *TLR* target lesion revascularization

### Rotational atherectomy in chronic total occlusion (CTO)

Although the inability to cross the lesion with a guidewire remains the most frequent cause for CTO PCI failure, lesions uncrossable or undilatable by a balloon due to severe calcification are not uncommon, and approaching uncrossable and/or undilatable CTOs in a systematic fashion can improve the success and safety of these procedures. Common steps to overcome these difficulties are to advance small low-profile balloons, special microcatheters and/or the “granadoplasty” technique (intentionally rupturing the balloon in the lesion), in combination with increasing guiding support using anchor balloon and guiding extension. Nevertheless, in some cases the use of RA or laser and if all else fails subintimal crossing techniques remain indispensable [56].

Whereas ROTAXUS excluded CTO lesions, in the PREPARE–CALC trial 2.5% of the treated lesions were CTOs. In general, RA is performed in 2.5% to 8.2% of CTO PCIs and is usually needed in patients with multiple comorbidities (advanced age, diabetes mellitus, previous CABG) having very complex CTO lesions with high J-CTO scores [57–63]. Published experience of RA in CTO lesions is shown in Tables 5 and S4.

**Table 5:** Published experience with RA in CTO lesions (important patient and lesion characteristics)

Author	Year	RA (%)	*n* (CTO)	Age (y)	DM (%)	CABG (%)	J-score	Antegrade (%)	Retrograde (%)	DRT (%)	Uncros (%)	Undil (%)	Bailout RA (%)
Xenogiannis et al., (PROGRESS CTO registry) [57]	2019	116 (3.3%)	3540	68 ± 8	61	48	3 ± 1.2 ≥ 2 (86.2%)	69	22	12	–	–	43.6
Brinkmann et al. [58]	2018	75 (2.5%)	2972	69.5 ± 8.5	32	32	2.7 ± 1.3 ≥ 2 (80.3%)	87	13	9.3	73.3	26.4	-
Huang et al. [59]	2017	26 (8.2%)	316	71.1 ± 12.7	50	15.3	≥ 2 (100%)	76.9	23.1	–	–	–	–
Azzalini et al. [60]	2016	35 (3.5%)	1003	68.9 ± 9.5	58	31	2.5 ± 1.1 ≥ 2 (80%)	74	6	20	51	49	77
Zhang et al. [62]	2016	26 (4.6%)	525	64 ± 8.1	30.8	–	2.9 ± 0.69	–	–	–	100	0	–
Pagnotta et al. [61]	2012	31 (4.1%)	748	67 ± 9	45	25	–	–	–	–	–	–	–
Pagnotta et al. [63]	2010	45 (5.2%)	855	70 ± 8	46.5	40	–	–	–	–	–	–	–

Data are presented in number or percentage, mean ± standard deviation or median [interquartile range]

*CABG* previous coronary artery bypass graft; *CTO* Chronic Total Occlusion; *DM* diabetes mellitus; *DRT* dissection re-entry technique; *RA* rotational atherectomy; *Uncros* uncrossable lesions; *Undil* undilatable lesions; *bailout* RA performed in bailout situation

The key step for successful RA is the passage of the RotaWire™. The first step should be an attempt to exchange the conventional guidewire with a microcatheter or a low-profile over-the-wire balloon. The microcatheter is placed distal to the CTO lesion, the original wire is removed and replaced with the RotaWire™ [58]. In cases, where the microcatheter could not cross, one should try to advance it as far as possible, and then manipulate the RotaWire™ carefully to find the micro-channel created by the 0.014 inch wire. An extra-support RotaWire™ is preferred to increase guiding support, but a floppy Rota wire is better for primary rotawiring due to the longer tip length. Multiple views on angiography should be done to ensure that the wire is located within the true lumen distal to the lesion before RA [62]. RA is usually successfully performed in CTO procedures with a 1.25 mm burr, and more than 80% of lesions require only one burr [56]. While RA is mostly performed during antegrade CTO recanalization, the rate of RA in retrograde CTO procedures varies in the published experience between 6 to 23% of cases. Although RA in the sub-intimal space is traditionally considered contra-indicated, Azzalini et al. reported 20% of RA after dissection-re-entry techniques during CTO PCI at four specialized centers [60]. In another analysis of CTO procedures from the PROGRESS–CTO registry, RA after dissection-re-entry was performed in 12% of cases [57], and this rate was 9.3% in the cohort of Brinkmann et al. who also showed that even in the presence of coronary dissection RA was not associated with higher risk [58]. Two case reports described RA carried out in the subintimal space, one after switching to the RotaWire™ using the tip-in technique through the microcatheter [64], and the other case was performed in subadventitial retrograde CTO PCI over an RG3 guidewire (Asahi, Japan) due to failure to switch to the RotaWire™ with the tip-in technique [65]. These highly advanced techniques are off-license due to high risk of vessel perforation, and should only be tried with high caution by experienced operators.

### Rotational atherectomy and bifurcation lesions

Treatment of bifurcation lesions is a growing challenge in contemporary PCI because of the technical complexity, higher risk of periprocedural complications and worse outcome compared to non-bifurcation lesions [66]. In addition to the difficulties related to lesion preparation and device delivery, the presence of coronary calcification represents an additional challenge in treating bifurcation lesions. Fujino et al. demonstrated that the presence of coronary calcification at the site of a bifurcation assessed by OCT is associated with a higher risk of side branch occlusion [67]. In this context, the main issues to provide optimal vessel patency are careful lesion preparation to “soften” the lesion, prevention of plaque/carina shifting and careful carina reconstruction [68]. In an as-treated subgroup analysis of bifurcation lesions from the PREPARE–CALC trial, more compromised side branches at the end of the procedure were found in patients treated with MB as compared to those treated with RA. Post-procedural levels of cardiac biomarkers were also significantly lower in the RA group [69]. This finding could be attributed to the atheroablative effect of RA that reduces the plaque volume and consequently the risk of carina shift during lesion preparation and after stenting. In certain cases, RA is needed in both main and side branch. Iannaccone et al. reported on an LAD/D1-bifurcation, where they first treated the LAD with a 2 mm burr, and then advanced a RotaWire™ and guiding-extension in the diagonal branch and performed RA with a 1.5 mm burr through the guiding extension, thus protecting the wire left in the LAD [70].

### RA and acute coronary syndrome

In the setting of an acute coronary syndrome (ACS), moderate to severe calcification is present in up to 32% of patients and is associated with unfavorable outcome due to higher incidence of target lesion revascularization, stent thrombosis and mortality [71, 72]. This justifies the need for optimal lesion preparation in an effort to optimize stent implantation and consequently patient outcome. Based on theoretical assumptions, thrombogenic states such as unstable lesions are classically considered a relative contraindication to RA. Nevertheless, in real life, the use of RA in the setting of ACS can be the last interventional option. In the last few years, many analyses reported the safety and efficacy of performing RA in the setting of ACS (Table 6) [73–76]. Iannaccone et al. compared in an analysis from the multicenter ROTATE registry 484 patients with non-ST-elevation ACS to 824 patients with stable coronary artery disease (SCAD) [74]. Of interest, no differences in RA indication (elective RA in about half of cases in both groups) and in RA technique (burr size and burr-to-artery ratio) was observed between the groups. In another analysis form the Bad Segeberg single-center registry comparing RA in 108 ACS (including 8 STEMIs) vs. 433 SCAD, the burr-to-artery ratio tended to be smaller in the ACS group [73]. In a retrospective study of the Polish National data set, Januszek et al. reported excellent outcome in 112 NSTEMI and 133 STEMI patients treated with bailout RA in the setting of ACS [75]. Angiographic success was high in all reported series (ranged between 93% and 97.6%) and comparable with SCAD patients. In addition, slow/no-flow phenomenon occurred in no more than 3.3% of cases with no significant difference compared with SCAD. Kübler et al. reported similar 1-year outcome of RA between ACS (42 patients) and SCAD (164 patients) [76]. On the opposite, with longer follow-up period and more patients, long-term follow-up in the ROTATE and Bad Segeberg registries of RA-ACS patients revealed higher incidence of cardiovascular events compared to patients with SCAD (32.4% vs. 24.2%, *p* < 0.01 in ROTATE registry; 39.9% vs. 22.4%, *p* = 0.02 in Bad Segeberg registry). Both analyses demonstrated that ACS presentation was an independent predictor of MACE at long-term follow-up. Of note, after propensity score matching, Iannaccone et al. found no difference in MACE occurrence between patients with ACS who did or who did not undergo RA. That being said, RA can be safely performed in the setting of ACS by experienced operators, and efforts should be done to optimize stent results and consequently long-term outcomes.

**Table 6:** Published experience with RA in ACS

Author	Year	ACS (n)	SCAD (n)	Age (years)	Angiographic success (%)	Slow/no-flow (%)	IH-death (%)	IH-MACE (%)	FU (m)	FU-death (%)	FU-TLR (%)	FU-TVR (%)	FU-MACE (%)
Allali et al. [73]	2018	108	433	73.0 ± 9	96.6	0.8	1.8	3.7	25	15.6	–	26.8	39.9
Kübler et al. [76]	2018	43	164	75 ± 10	93	0	4.7	27	12	16.3	–	–	25.6
Iannaccone et al. (ROTATE registry)[70]	2016	484	824	71.4 ± 9.6	97.6	3.3	1.2	6.8	27.9	9.7	13.5	–	32.4
Januszek et al. (ORPKI registry)[75]	2018	245	530	73.1 ± 10.4	96.7*	3.3	0	2**	–	–	–	–	–

Data are presented in number or percentage, mean ± standard deviation

*ACS* acute coronary syndrome; *SCAD* stable coronary artery disease; *IH* in-hospital; *MACE* major adverse cardiovascular events; *FU* follow-up; *TVR* target vessel revascularization; *ORPKI* is the Polish Cardiovascular Intervention Society national registry; *RA* rotational atherectomy; *ROTATE* is the ROTathional AThErectomy registry conducted by eight centers in Italy

*Angiographic success was defined only according to TIMI 3 flow at the end of the procedure

**Rate of overall complications

### Rotational atherectomy and calcified nodules

Calcified nodule (CN) is pathologically defined as an eruptive accumulation of small nodular calcifications [77–79]. Pathology studies showed that CN causes 2% to 7% of fatal coronary thromboses [77–79]. OCT studies reported CN in 2 to 8% of patients presenting with ACS, and represents after calcific sheet the second most frequent cause of ACS in patients with severe calcified culprit lesions [80–83]. CN are mostly located in the RCA and have a higher calcification index compared to other calcified plaques [81, 83].

The role of RA in the treatment of CN is unclear. Recently, Watanabe et al. fond no difference in terms of 1-year ischemia-driven TVR of intravascular ultrasound detected CN in patients treated with RA compared to a matched population treated without RA. This study was limited by the relatively small number of patients (42 patients in each group) [84]. In another study, Morofuji et al. evaluated the impact of CN on outcomes of patients undergoing RA. In this analysis, patients with CN (128 patients) had higher incidence of 5-year clinically driven TLR and stent thrombosis compared to patients without CN (136 patients) [85]. To improve stent results more aggressive lesion preparation such as RA in combination with orbital atherectomy [86] or RA with cutting balloon or intravascular lithotripsy might be necessary. However, because of unstable features and heterogeneous hardness of CN, distal embolization of CN can occur after RA [87].

### RA and impaired left ventricular function

RA was historically not recommended in patients with reduced left ventricular ejection fraction (LVEF) due to inherent acute cardio-depressive effects of the burr runs [88]. Contemporary RA practice is assumed to cause less vessel injury and could, therefore, be a safe strategy in patients with impaired LVEF [3].

In a study by McEntegart et al. RA was associated with a type 4a MI rate of 24% as detected by cardiac magnetic resonance [89]. In two other studies by Watanabe et al. [90] and Whiteside et al. [91], slow flow was significantly higher in severely-reduced LVEF patients (defined as ≤ 35% by Watanabe et al. and as ≤ 30% by Whiteside et al.). A recent study found slow flow to be more than fivefold higher in patients with severely reduced LVEF (≤ 35%) as compared to preserved LVEF (≤ 55%) [92]. In another analysis done by Mankerious et al. patients with severely reduced LVEF had worse acute outcome after RA as well as higher mortality on long term [92].

Although not specifically addressed by contemporary practice guidelines, hemodynamic support would theoretically convey an extra benefit in patients with reduced LVEF undergoing RA, where the hemodynamic consequences of intervention are more profound than standard PCI. In a sub-analysis of the PROTECT II trial (A Prospective, Multicenter Randomized Controlled Trial of the IMPELLA RECOVER LP 2.5 System vs. Intra Aortic Balloon Pump [IABP] in Patients Undergoing Non Emergent High Risk PCI), patients treated with RA under Impella 2.5 l/min received more aggressive lesion preparation with RA (longer duration, more passes per lesion and more LMT lesions) resulting in more periprocedural myocardial infarctions but fewer revascularization events at 90 day follow-up as compared with those treated under IABP [93]. Nevertheless, this analysis was limited by its small sample size (32 patients with Impella 2.5 l/min vs. 20 patients with IABP). In summary, patients with severely reduced ejection fraction treated with RA are more likely to develop peri-procedural events such as slow–flow and MI. Use of hemodynamic support might offer operators the comfort to obtain optimal lesion preparation and stent results. Whether this practice improves in-hospital outcome remains to be determined.

## RA combined with other calcium treatment strategies

### RA plus cutting balloon

OCT studies revealed that plaque modification with RA is typically a polished, concave, round shape of the surface of calcium. Together with calcium fracture and plaque redistribution achieved with additional balloon dilatation, these plaque modifications improve lesion compliance to allow more uniform stent expansion. A conventional balloon tears the arterial wall at the weakest point (usually at the junction of normal with the diseased segment), whereas cutting balloon (CB), which has three-to-four longitudinal blades mounted on the outer surface, creates controlled incisions in the plaque.

Amemiya et al. found that the number and thickness of calcium fractures as assessed by OCT were greater and the stent expansion was better when CB was used after RA compared with conventional balloons [94]. In the same direction, Li et al. in a pilot randomized trial comparing 35 patients treated with RA in combination with CB vs. 36 RA patients, showed that aggressive plaque preparation with RA plus CB seems to be safe and results in larger acute cross-sectional area gain after stenting in an IVUS analysis. Acute lumen gain tended to be higher and TLR after 1 year was significantly lower in the RA plus CB group [95]. In the ongoing PREPARE–CALC COMBO trial the combination of RA with CB followed by Orsiro DES implantation will be historically compared with both arms of the PREPARE–CALC trial (Clinicaltrials.gov: NCT04014595).

### RA and lithotripsy

In case of thick calcium particularly when deeply located in the intimal and medial layers, atherectomy (rotational or orbital) may result in eccentric ablation with no effect on deeper calcium and subsequent sub-optimal lesion dilatation associated with potential stent under-expansion. Lithoplasty is a trans-catheter technique based on ultrasound shock waves, in which multiple emitters mounted on a traditional balloon catheter platform create diffusive, circumferential pulsatile pressure waves up to 50 atm aiming to disrupt calcified plaque. Following intra-vascular lithotripsy (IVL), circumferential calcium fractures (by intimal and medial calcification disruption) have been demonstrated in OCT [96].

Safety and efficacy of the complementary use of RA and lithotripsy were reported in two case series and some case reports [97–100]. Using the two techniques together provides the following advantages: RA (by intimal calcium ablation) to enable initial lesion modification and ease the passage of balloon catheters, and IVL (by intimal and medial calcium breakage) to allow the final successful dilatation of heavily calcified lesions. The planed Rota-Tripsy combination seems to be a reasonable approach in case of deep and superficial calcium distribution revealed by pre-procedural intravascular imaging. Moreover, Rota-Tripsy could be used as a bailout strategy to compensate the disadvantage of each technique alone: in case of balloon-undilatable lesions after RA, or to enable the advancement of IVL balloon (with poor crossing profile) in long calcified lesions. A case report described the safe off-label use of IVL to improve stent-expansion in an RA-resistant calcified plaque [101]. The Rota-Tripsy combination has also been performed successfully to treat in stent restenosis due to calcified neoatherosclerosis [102]. Due to lacking of data in this field, prospective and randomized clinical trials are required to define the efficacy of this approach particularly in comparison with other calcium modifying techniques and to determine long-term outcomes after this costing and aggressive lesion preparation.

### RA and laser atherectomy

Excimer laser coronary atherectomy (ELCA) is currently indicated for the treatment of mild to moderate calcified lesions resistant to balloon inflation, saphenous venous grafts and in-stent restenosis [103]. The combined use of RA and ELCA is particularly effective for heavily calcified non-crossable, non-dilatable lesions. The utility of ELCA comes when the RotaWire™ could not be delivered into the distal coronary vessel. In this situation, ELCA can be used to modify the lesion and create a channel through which a RotaWire™ can subsequently be delivered distally. This is useful in CTO cases when no equipment can cross the lesion. ELCA can also be used in cases of underexpanded stents even after atherectomy and maximum balloon dilation [104].

## Practical approach to treat calcified coronary lesions

Intracoronary imaging is one cornerstone to plan or guide the procedure as fluoroscopy has limitations in detection of coronary calcification [20]. However, it is frequent that calcified complex lesions are not crossable by imaging catheters and angiographically invisible calcification does not appear to inhibit stent expansion [105]. On the other hand, severely calcified lesions with radio-opacities on both sides of the vessel wall [106] deserve aggressive lesion preparation regardless of the additional information obtained from intracoronary imaging. Furthermore, when comparing different lesion preparation techniques, no difference was found concerning acute lumen gain and stent expansion between RA and MB in the PREPARE–CALC trial [4, 22]. Similarly, in the recently published ISAR–CALC trial, lesion preparation with super high-pressure balloon vs. scoring balloon led to a comparable stent expansion index (0.72 ± 0.12 vs. 0.68 ± 0.13; *p* = 0.22) [107].

Therefore, from a practical point of view, bail-out RA should be avoided in patients with angiographically severe coronary calcification, as this setting prolongs the procedure and is associated with unfavorable acute outcomes [41]. We suggest to first evaluate whether the lesion is complex with the previously described characteristics, namely, type C (particularly angulated and long lesions), very tight (> 80%) and bifurcation lesions with high risk of side branch compromise. If the lesion is complex, an upfront RA should probably be the default strategy. If the angiographic characteristics do not correspond to the mentioned characteristics, we suggest a balloon-based strategy with either cutting/scoring balloons, OPN balloons, or intravascular lithoplasty.

**Fig. 5:**
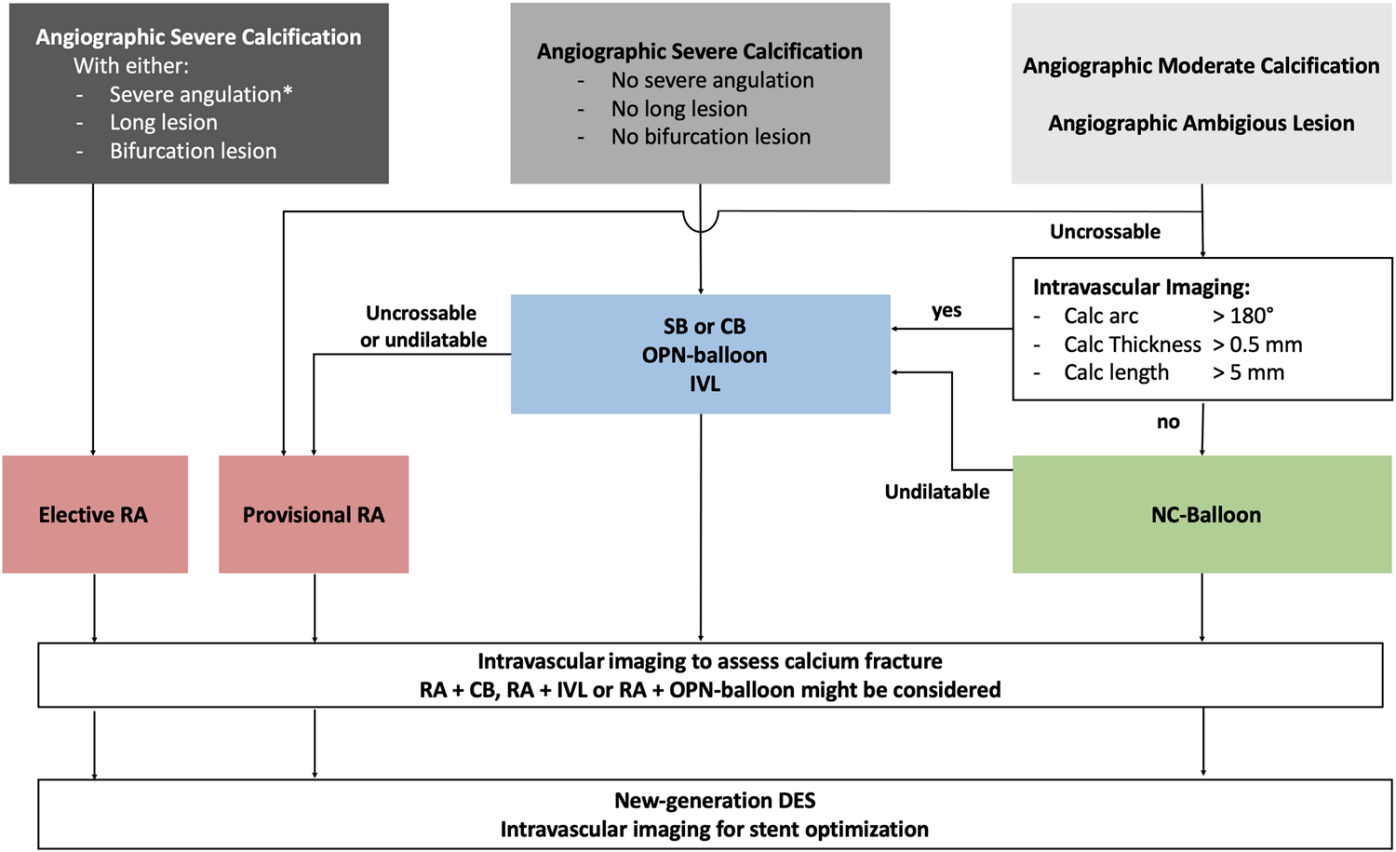
Practical approach to treat calcified coronary lesions. *Use of floppy RotaWire™ and small burr are recommended to avoid complications by wire bias. *CB* cutting balloon; *DES* drug-eluting stents; *IVL* intra-vascular lithotripsy; *NC-Balloon* non-compliant balloon; *OPN-Balloon* super high-pressure balloon; *RA* rotational atherectomy

In case of moderate calcification or ambigious lesions, intravascular imaging should be considered to plan the procedure [108]. Fujino et al. developed an OCT-based score to predict stent underexpansion [109]. A calcific arc of more than 180°, calcific plaque thickness of more than 0.5 mm and calcific plaque length of more than 5 mm is considered to prevent adequate stent expansion. In this case, we recommend the use of a balloon-based approach with aggressive preparation potential, such as IVL, CB or OPN balloon. If calcific plaque extent is not that high, the use of a non-compliant balloon should be sufficient to achieve optimal lesion preparation (Fig. 5). After RA, 1-to-1 balloon angioplasty using non-compliant balloon is done before stent implantation and a full balloon expansion should be achieved.

In case of large calcium burden or suspicion of insufficient lesion compliance after initial lesion preparation, intravascular imaging might be considered. In absence of calcium fracture [110] more aggressive lesion preparation (combination of RA + CB, or RA + IVL) should be performed. Finally, intravascular imaging after stent implantation should be systematically done to optimize final results.

## Conclusions

With the growing experience, technical improvement and new-generation DES use, recent studies showed satisfactory acute and long-term results after RA in calcified coronary lesions. In this review article we illustrated the evolution of RA use in its technique and indications. From a technical point of view, the currently used technique are based on less aggressive debulking with more effort to optimize stent results using complementary balloon optimization and intravascular imaging. The combination with modern sophisticated tools and improved operator skills allows performing successful PCI in very difficult situations. As for current indications, a rational and selective rotablation should be the preferred approach to safely and efficiently accomplish challenging PCI cases. Although encouraging acute procedural data have been reported in very high-risk patients after RA, efforts are still needed to optimize long-term outcome.

## Supplementary Information

Below is the link to the electronic supplementary material.

Supplementary file1 (DOCX 38 kb)

## Data Availability

Data analysis was performed in the Heart Center Segeberger Kliniken GmbH and data are available.

## Abbreviations

CB: Cutting balloon
IVL: Intra-vascular lithotripsy
MB: Modified balloon
RA: Rotational atherectomy
ROTAXUS: Rotational atherectomy prior to taxus stent treatment for complex native coronary artery disease
PREPARE–CALC: Comparison of strategies to prepare severely calcified coronary lesions
SB: Standard balloon
