# Supplementary material for: Rotational atherectomy of calcified coronary lesions: current practice and insights from two randomized trials
Source: Clin Res Cardiol. 2022 Apr 28;112(9):1143–63. doi: 10.1007/s00392-022-02013-2 (PMC10450020; doi:10.1007/s00392-022-02013-2)
Supplement: Supplementary file 1 — Supplementary file1 (DOCX 38 kb) [file 392_2022_2013_MOESM1_ESM.docx]

Supplemental Material

Table S1: Baseline characteristics (n=220 patients)

|  | PREPARE-CALC (n = 100) | ROTAXUS (n = 120) | p-value |
| --- | --- | --- | --- |
| Age (years) | 74.3±7.1 | 70.5±8.2 | 0.0003 |
| Males | 77 (77%) | 86 (72.3%) | 0.37 |
| BMI (kg.m^-2^) | 28.0±5.1 | 27.9±4.3 | 0.94 |
| Diabetes mellitus | 34 (34%) | 32 (26.8%) | 0.24 |
| Hypertension | 93 (93%) | 106 (89.1%) | 0.31 |
| Dyslipidemia | 68 (68%) | 91 (76%) | 0.16 |
| Current smokers | 15 (15%) | 24 (20.2%) | 0.32 |
| Chronic renal failure* | 26 (26%) | 17 (14.2%) | 0.03 |
| Previous MI | 21 (21%) | 38 (31.9%) | 0.07 |
| Previous PCI | 47 (47%) | 44 (37%) | 0.15 |
| Previous CABG | 6 (6%) | 9 (7.6%) | 0.64 |
| Unstable angina | 8 (8%) | 17 (14.2%) | 0.15 |
| Atrial fibrillation | 18 (18%) | 13 (11%) | 0.18 |
| Left main disease | 23 (23%) | 11 (9.2%) | 0.005 |
| Multivessel disease | 74 (74%) | 88 (74%) | 0.99 |
| LV ejection fraction (%) | 55.7±11.7 | 55.5±10.6 | 0.91 |
| Multilesion PCI | 35 (35%) | 23 (19.3%) | 0.009 |
| Unfractionated heparin | 99 (99%) | 49 (41.2%) | <0.001 |
| Bivalirudin | 1 (1%) | 70 (58.8%) | <0.001 |
| GP IIb/IIIa antagonists | 2 (2%) | 4 (3.4%) | 0.54 |

Values are n (%) or mean ± SD;

BMI= body mass index, CABG=coronary artery bypass graft, GP=glycoprotein, LV=left ventricle, MI=myocardial infarction, PCI=percutanous coronary intervention.

*defined as glomerular filtration rate < 60 ml/min

Table S2: Quantitative coronary angiography data (n=283 lesions)

|  | PREPARE-CALC  (n = 137) | ROTAXUS (n = 146) | p-value |
| --- | --- | --- | --- |
| Before procedure |  |  |  |
| Lesion length (mm) | 20.86±12.30 | 19.59±9.66 | 0.35 |
| Reference vessel diameter (mm) | 3.10±0.49 | 2.68±0.41 | <0.001 |
| Minimal lumen diameter (mm) | 1.15±0.35 | 1.01±0.36 | 0.002 |
| Diameter stenosis (%) | 63.43±9.80 | 62.23±12.01 | 0.30 |
| Severe calcification* | 104 (76.05%) | 65 (44.5%) | <0.001 |
| Immediately after procedure |  |  |  |
| Minimal lumen diameter (mm) |  |  |  |
| In-stent | 2.85±0.43 | 2.57±0.38 | <0.001 |
| In-segment | 2.62±0.67 | 2.26±0.49 | <0.001 |
| Diameter stenosis (%) |  |  |  |
| In-stent | 12.62±5.36 | 10.79±5.61 | 0.001 |
| In-segment | 17.58±7.31 | 18.21±8.77 | 0.92 |
| Acute gain (mm) |  |  |  |
| In-stent | 1.70±0.42 | 1.56±0.43 | 0.017 |
| In-segment | 1.47±0.64 | 1.24±0.54 | 0.006 |
| Re-angiography at 9 months |  |  |  |
| Late lumen loss  In-stent  In-segment | 0.22±0.39  0.18±0.74 | 0.44±0.58  0.39±0.57 | 0.002  0.04 |

Values are n (%) or mean ± SD

*as adjudicated by the angiographic corelab

Table S3: Procedural and in-hospital outcome (n=220 patients)

|  | PREPARE-CALC  (n = 100) | ROTAXUS (n = 120) | p-value |
| --- | --- | --- | --- |
| Procedural duration (min) | 88.2±34.9 | 66.4±44.5 | <0.001 |
| Fluoroscopy time (min) | 23.9±12.2 | 22.8±21.9 | 0.28 |
| Contrast amount (ml) | 233.0±109.1 | 201±113.6 | 0.002 |
| Procedural outcome |  |  |  |
| Large dissection (> 5mm) | 3 (3%) | 4 (3.3%) | 0.88 |
| Perforation | 4 (4%) | 2 (1.7%) | 0.41 |
| Pericardial effusion | 3 (3%) | 5 (4.2%) | 0.73 |
| No/slow flow | 2 (2%) | 0 (0%) | 0.20 |
| Stent failure | 1 (1%) | 0 (0%) | 0.46 |
| Crossover | 0 (0%) | 5 (4.2%) | 0.038 |
| Strategy success* | 98 (98%) | 111 (93%) | 0.11 |
| In-hospital outcome |  |  |  |
| Death | 0 (0%) | 2 (1.7%) | 0.50 |
| Myocardial infarction | 2 (2%) | 2 (1.7%) | 1.00 |
| Target vessel re-PCI | 0 (0%) | 1 (0.8%) | 1.00 |
| CABG | 0 (0%) | 1 (0.8%) | 1.00 |
| Stent thrombosis | 0 (0%) | 0 (0%) | 1.00 |
| Access site complications | 3 (3%) | 7 (5.9%) | 0.31 |

Values are n (%) or mean ± SD;

CABG=coronary artery bypass graft; PCI=percutaneous coronary intervention;

Table S4: Published experience with RA in CTO lesions. In-hospital and long-term outcome.

| Author | Success (%) | Periprocedural outcome (%) | Follow (months) | Long-term outcome (%) |
| --- | --- | --- | --- | --- |
| Xenogiannis et al (PROGRESS CTO resgistry)[1] | 90 | MACE 4  Perforation 10  Donor vessel injury 4  Tamponade 2.6 | - | - |
| Brinkmann et al,[2] | 94.7 | Perforation 1.3  Decompensation1.3 | - | - |
| Huang et al,[3] | 89.5 | MACE 3.8  MI 0  TLR 0  CV death 0 | 38.4 | MACE 12  MI 7.7  TLR 3.8  CV death 3.8 |
| Azzalini et al,[4] | 77 | Slow flow 17  Bradyarrhythmia 17  Dissection 29 | 21.9 | MACE 15  Death 6  TV MI 9  TVR 6 |
| Zhang et al,[5] | 96.15 | MACE 0 | - | - |
| Pagnotta et al,[6] | 84 | MI* 29 | - | - |
| Pagnotta et al,[7] | 95.5 | MI* 35 | - | - |

Data are presented in number or percentage.

MACE = Major adverse cardiovascular events, MI = Myocardial infarction, TLR = Target lesion revascularization, TV MI = Target Vessel myocardial infarction, TVR = Target vessel revascularisation.

*Peri-procedural myocardial infarction (defined as a creatinine-kinase MB increase >3 ULN)

*References*

1. Xenogiannis I, Karmpaliotis D, Alaswad K, Jaffer FA, Yeh RW, Patel M, Mahmud E, Choi JW, Burke MN, Doing AH, Dattilo P, Toma C, Smith AJC, Uretsky B, Krestyaninov O, Khelimskii D, Holper E, Potluri S, Wyman RM, Kandzari DE, Garcia S, Koutouzis M, Tsiafoutis I, Khatri JJ, Jaber W, Samady H, Jefferson BK, Patel T, Moses JW, Lembo NJ, Parikh M, Kirtane AJ, Ali ZA, Doshi D, Tajti P, Rangan BV, Abdullah S, Banerjee S, Brilakis ES (2019) Usefulness of Atherectomy in Chronic Total Occlusion Interventions (from the PROGRESS-CTO Registry). Am J Cardiol 123 (9):1422-1428. doi:10.1016/j.amjcard.2019.01.054

2. Brinkmann C, Eitan A, Schwencke C, Mathey DG, Schofer J (2018) Rotational atherectomy in CTO lesions: too risky? Outcome of rotational atherectomy in CTO lesions compared to non-CTO lesions. EuroIntervention 14 (11):e1192-e1198. doi:10.4244/EIJ-D-18-00393

3. Huang WC, Teng HI, Chan WL, Lu TM (2018) Short-term and long-term clinical outcomes of rotational atherectomy in resistant chronic total occlusion. J Interv Cardiol 31 (4):458-464. doi:10.1111/joic.12489

4. Azzalini L, Dautov R, Ojeda S, Serra A, Benincasa S, Bellini B, Giannini F, Chavarria J, Gheorghe LL, Pan M, Carlino M, Colombo A, Rinfret S (2017) Long-term outcomes of rotational atherectomy for the percutaneous treatment of chronic total occlusions. Catheter Cardiovasc Interv 89 (5):820-828. doi:10.1002/ccd.26829

5. Zhang B, Wang F, Tan JWC, Liao H, Chai W, Yu H, Yan H, Jin L (2016) The Application of Rotational Atherectomy in PCI of Coronary Chronic Total Occlusions. ASEAN Heart J 24:1. doi:10.7603/s40602-016-0001-8

6. Pagnotta P, Briguori C, Ferrante G, Visconti G, Focaccio A, Belli G, Presbitero P (2013) Tornus catheter and rotational atherectomy in resistant chronic total occlusions. Int J Cardiol 167 (6):2653-2656. doi:10.1016/j.ijcard.2012.06.124

7. Pagnotta P, Briguori C, Mango R, Visconti G, Focaccio A, Belli G, Presbitero P (2010) Rotational atherectomy in resistant chronic total occlusions. Catheter Cardiovasc Interv 76 (3):366-371. doi:10.1002/ccd.22504
